# Transcriptional profiling of innate and adaptive human immune responses to mycobacteria in the tuberculin skin test

**DOI:** 10.1002/eji.201141841

**Published:** 2011-07-29

**Authors:** Gillian S Tomlinson, Tamaryn J Cashmore, Paul T G Elkington, John Yates, Rannakoe J Lehloenya, Jhen Tsang, Michael Brown, Robert F Miller, Keertan Dheda, David R Katz, Benjamin M Chain, Mahdad Noursadeghi

**Affiliations:** 1 Infection and Immunity, University College LondonLondon, UK; 2 Lung Infection and Immunity Unit, Division of Pulmonology and Clinical Immunology, Department of Medicine, University of Cape TownCape Town, South Africa; 3 Department of Infectious Diseases, Imperial CollegeLondon, UK; 4 Hospital for Tropical DiseasesLondon, UK; 5 Department of Clinical Research, London School of Hygiene and Tropical MedicineLondon, UK; 6 Centre for Sexual Health and HIV Research, University College LondonLondon, UK; 7 Institute of Infectious Disease and Molecular Medicine, University of Cape TownCape Town, South Africa

**Keywords:** Delayed-type hypersensitivity, Gene expression profiling, Innate immunity, Th1 type responses, Tuberculin skin test

## Abstract

The tuberculin skin test (TST) is a model of integrated innate and adaptive human immune responses to *Mycobacterium tuberculosis*, but the component processes that are involved in this model have not previously been defined in vivo. We used transcriptional profiling to study these responses within the TST at molecular and system levels. Skin biopsies from TST injection sites were examined in subjects classified as TST^+^ or TST^−^ by clinical and histological criteria. Genome-wide expression arrays showed evolution of immune responses reflecting T-cell activation and recruitment with uniquely Th1-polarized responses and cytotoxic T cells (CTLs). In addition, distinct innate immune and IFN-γ-stimulated gene expression signatures were identified, under the regulation of NF-κB and STAT1 transcriptional control. These were highly enriched for chemokines and MHC class II molecules providing a potential mechanism for paracrine amplification of inflammatory responses in the TST, by supporting cellular recruitment and enhancing antigen presentation. The same repertoire of innate and adaptive immune responses was evident in TST^+^ and TST^−^ subjects alike, clinically positive TSTs being distinguished only by quantitatively greater differences. These data provide new insights into complex multifaceted responses within the TST, with much greater sensitivity than previous clinical or histological assessments.

## Introduction

The current understanding of host defense against mycobacteria and the immunopathogenesis of tuberculosis (TB) is modelled on delayed-type hypersensitivity (DTH). This is a paradigm of complex immune responses at the interface of innate and adaptive immunity. In this model, innate immune cellular activation of mononuclear phagocytic cells leads to recruitment of circulating immune cells and activation of T cells bearing cognate T-cell receptors. In the presence of appropriate co-stimulatory signals, Th1 type responses associated with production of IFN-γ and consequently IFN-γ-stimulated processes follow. This component is an important factor in local paracrine amplification of the inflammatory response and activation of cell-mediated antimicrobial killing.

However, our understanding of the interface between innate and adaptive human immune responses relies heavily on in vitro or ex vivo models. In vivo studies of the molecular components of complex immunological processes within the tissue microenvironment are needed to test these models in man. A classic paradigm of DTH, which might be amenable to such an approach, is represented by the tuberculin skin test (TST). The TST is regarded as an index of the presence of T-cell memory for mycobacterial antigens in the clinical assessment of TB. However, considerable discordance between TSTs and quantitation of anti-mycobacterial T-cell memory in peripheral blood is recognized 1. For example, deficient DTH responses are seen in the skin of elderly subjects or amongst HIV co-infected patients, despite preserved peripheral blood T-cell responses 2–4. Although the TST has been assessed by histology and immunostaining previously 2, 5, 6, many questions remain unresolved. In particular, little is known about the innate immune and IFN-γ-stimulated responses in the TST. Similarly, our understanding of the mechanisms that underlie TST anergy, which is known to arise in elderly patients with TB, patients with miliary TB or with TB/HIV co-infection, and in patients with sarcoidosis, is incomplete. To obtain new insights into the immunological features of the TST at a molecular level, we performed transcriptional profiling of skin biopsy samples from subjects categorized as TST^+^ and TST^−^ by conventional clinical and histological criteria. We focussed on gene expression signatures for innate immune responses, differential T-cell responses, and responses stimulated by IFN-γ.

Our hypothesis was that early innate immune gene expression would be independent of T-cell memory for mycobacterial antigens, and would be augmented only in the presence of Th1 responses accompanied by molecular evidence for IFN-γ-stimulated gene expression. Here, we present findings that identify innate and IFN-γ-stimulated gene expression signatures in samples from TST^+^ subjects, and show evidence for T-cell activation, Th1-polarized responses and CTLs, but not for cell proliferation, implying that immune responses were predominantly due to cell recruitment. Unexpectedly, however, identical gene expression patterns were observed in subjects judged to be TST^−^ on conventional criteria, and samples from TST^+^ subjects were distinguished only by quantitative differences.

## Results

### Microarray gene expression profiles in early and late TST biopsies

**Figure 1:**
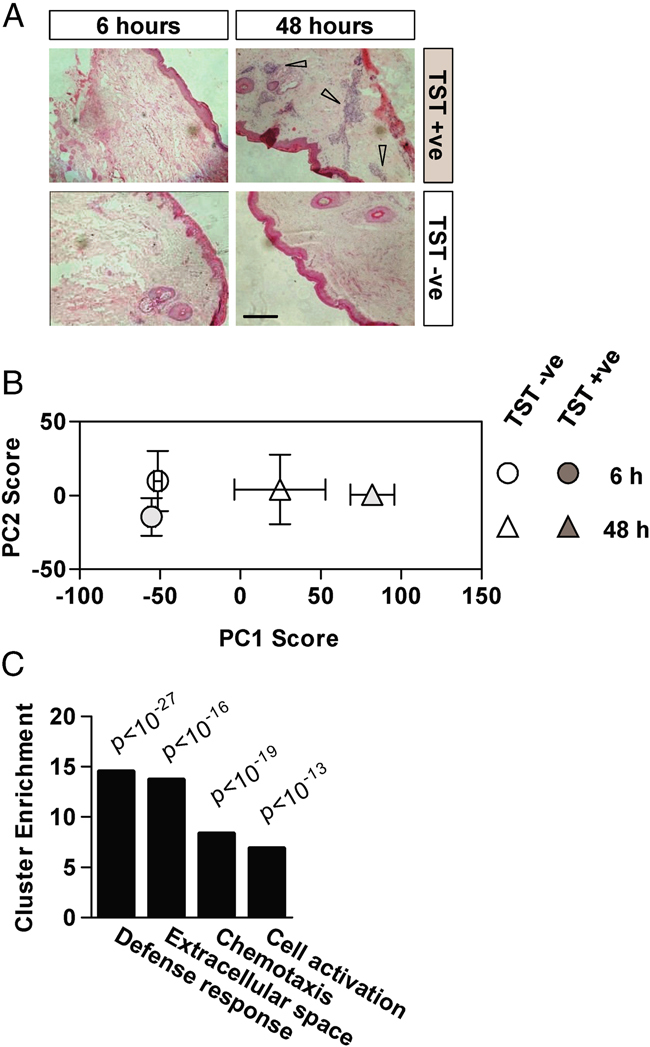
Transcriptional profiling of the TST. TST site skin biopsies from all 20 participants (40 biopsies) were stained with haematoxylin and eosin for histological analysis. (A) Representative histology from 6- and 48-h TST samples is shown. Arrows indicate areas of prominent intradermal inflammatory infiltrate in a localized perivascular distribution. No inflammatory infiltrate was seen in 6-h biopsy samples or in the 48-h samples from TST^−^ subjects (scale bar=400 μM). (B) Principle component analysis (PCA) of microarray data from TST^+^ and TST^−^ individuals (data points represent mean±SEM of PC scores from four separate subjects in each group). (C) Functional annotation clustering of the 250 genes which displayed the largest expression changes within PC1 was performed to show significantly enriched gene ontology groups (Modified Fisher's Exact Test).

Healthy HIV-1^−^ volunteers from the UK and South Africa (SA), representing an ethnically diverse study sample with mixed exposure history to mycobacteria and BCG vaccination, were subjected to concurrent TSTs in each arm and skin biopsies for histology and transcriptional profiling at 6 and 48 h. Clinical and histological classification of 48-h TST skin biopsy samples (Fig. 1A) was concordant in every instance apart from one SA subject who was clinically negative, but had histological inflammation, and was therefore included in the TST^+^ group for subsequent analysis. Demographic data, clinical measurements of the TST response and results of peripheral blood IFN-γ release assays (IGRA) are summarized in Supporting Information Table 1.

Genome-wide transcriptional profiles of 6 and 48-h samples from four TST^+^ subjects and four TST^−^ subjects, with the best RNA quality and equal representation from UK and SA groups (indicated in Supporting Information Table 1), were compared by principle component analysis (PCA). In this approach to represent multidimensional gene expression data, principle component (PC) scores reflect the magnitude of differences and each component reflects different dimensions of the gene expression data. The component of the data responsible for the greatest variance, denoted principle component (PC)1, clearly showed differential clustering of gene expression profiles of 48-h biopsies from TST^+^ and TST^−^ subjects, and from all 6-h biopsies, which clustered together (Fig. 1B). Functional annotation clustering analysis of the gene list that generated the top 10% of the total variance, identified from the PCA (factor) loadings in PC1, showed highly significant enrichment for gene ontology clusters involving immune responses (Fig. 1C). These data confirm that gene expression profiling may be used to assess evolution of complex immune responses in vivo within the TST. Evolution of immunological responses in 48-h samples from TST^−^ subjects was also evident, suggesting that gene expression profiling provides more sensitive assessment of the TST than previous clinical or histological studies. The finding that these gene expression changes occur in the same PC as those of 48-h samples from TST^+^ subjects suggests that immune responses in TST^−^ subjects are qualitatively similar, but quantitatively less than those of TST^+^ subjects.

### T-cell gene expression profiles in TSTs

**Figure 2:**
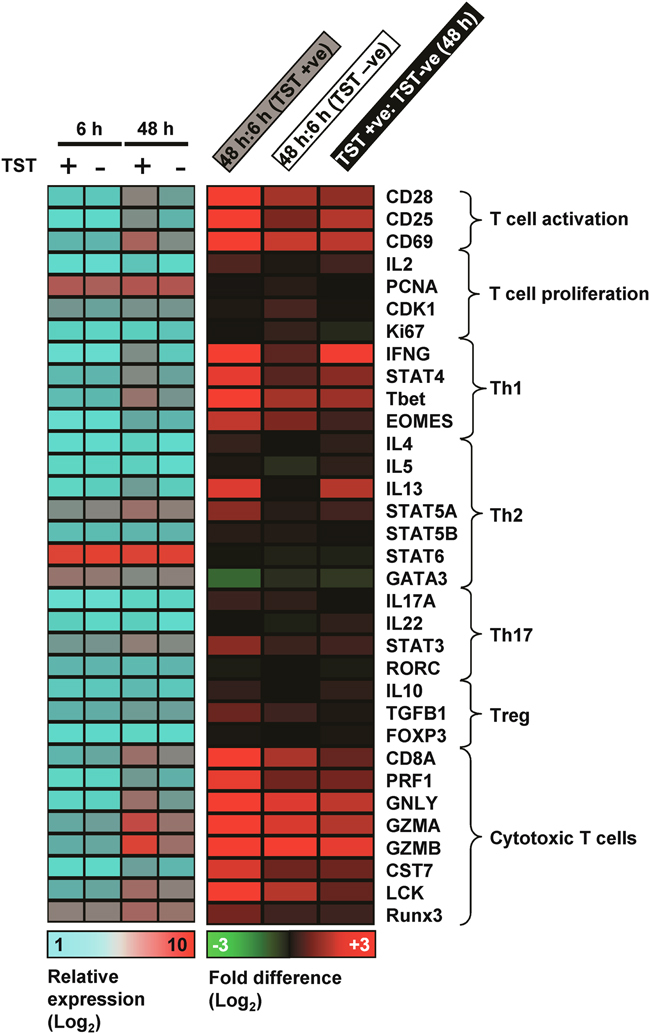
T-cell gene expression signatures in the TST. Transcriptional responses of gene sets representative of T helper and cytotoxic T-cell subsets as well as T-cell activation and proliferation markers are summarized by alignment with expression matrices showing either relative expression or mean fold differences in 6-h compared with 48-h biopsies from TST^+^ and TST^−^ subjects. Data represent mean gene expression values of four samples in each group.

Th1 responses are thought to be important determinants of DTH and immune responses to *Mycobacterium tuberculosis* (Mtb), but the roles of alternatively polarized Th subsets, T regulatory (Treg) cells and CTLs (CD8^+ve^) have not been established in vivo. Therefore, we used selected gene expression profiles reflecting T-cell subsets 7, 8 together with a panel of T-cell activation and proliferation markers, to probe transcriptional profiles in TST samples (Fig. 2). Expression of genes associated with T-cell activation, Th1 responses and CTLs was higher (>two-fold) in 48-h biopsies compared to 6-h biopsies and in 48-h samples from TST^+^ compared to TST^−^ subjects. In contrast, there was no consistent difference in expression of genes associated with T-cell proliferation, Th2 or Th17-polarized responses or Treg cells. Thus, the 48-h TST is associated with uniquely Th1-polarized responses and CTL gene expression profiles that were evident in samples from both TST^−^ and TST^+^ subjects, albeit with lower gene expression levels in TST^−^ subjects. Of note, our data showed no detectable increase in expression of natural killer cell markers, such as CD56, CD57 or killer cell immunoglobulin-like receptors, that may also generate IFN-γ responses (not shown), supporting the conclusion that increased IFN-γ gene expression reflected Th1 activity.

### Innate immune and IFN-γ-stimulated gene expression signatures in the TST

**Figure 3:**
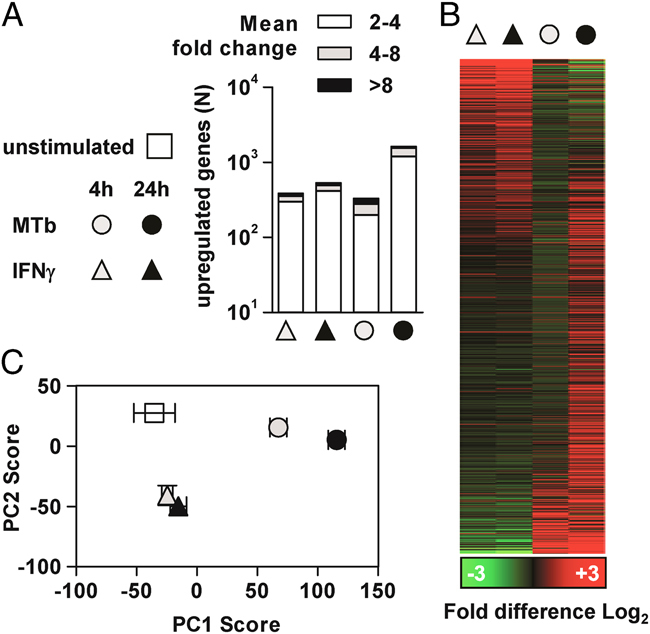
Discrimination of innate immune and IFN-γ-stimulated gene expression signatures in monocyte-derived macrophages. (A) Upregulation of gene expression in monocyte-derived macrophages (MDMs) stimulated for 4–24 h with Mtb (as an innate immune stimulus) or with 10 ng/mL IFN-γ. Three separate experiments from different donors for each stimulation group were compared with eight separate experiments from different donors for unstimulated MDMs. (B) All significant gene expression differences were aligned in an expression matrix representing mean fold change compared with unstimulated MDMs. (C) The differences in transcriptional profiles from unstimulated MDMs and Mtb or IFN-γ-stimulated MDMs were also assessed by PCA (data points represent mean±SEM of PC scores).

**Figure 4:**
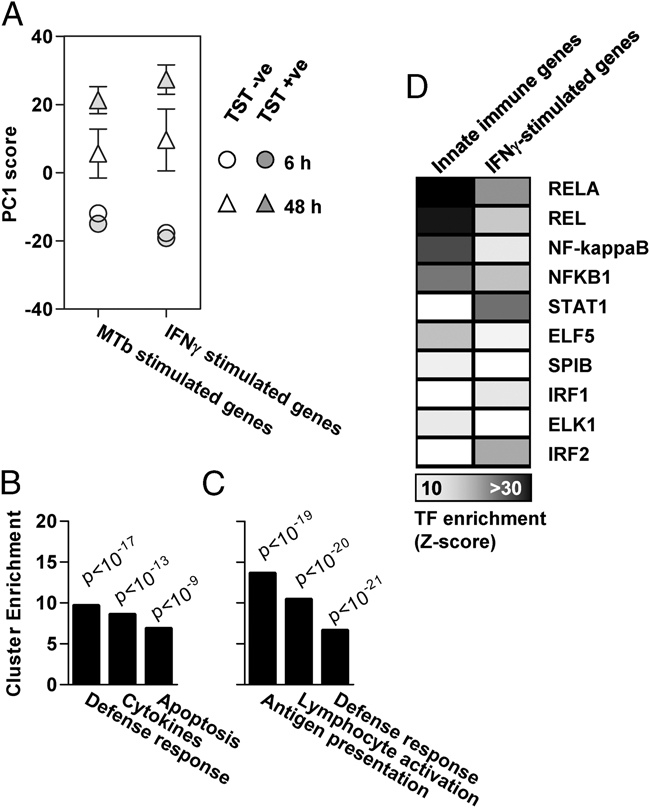
Innate immune and IFN-γ-stimulated gene expression signatures in the TST. (A) The major differences identified by PCA and represented by the PC1 score in transcriptional profiles from TST arrays in TST^+^ and TST^−^ subjects were compared for Mtb or IFN-γ-stimulated gene lists (data points represent mean±SEM of PC scores from four separate subjects in each group). Among genes that showed increased expression in 48-h TST samples (>two-fold over 6-h samples), the top three significantly enriched gene ontology associations are shown, in either (B) innate immune or (C) IFN-γ-stimulated gene expression signatures (Modified Fisher's Exact Test). (D) These in vivo signatures were subjected to transcription factor binding site enrichment analysis to determine the putative transcriptional regulators of each response. In this analysis, *z-*scores of >10 are considered to indicate highly significant over-representation of transcription factor binding sites within the analysed gene list.

Although simple observational studies suggest that the TST response involves interactions between mononuclear phagocytes and T cells, the TST has been interpreted almost exclusively as a reflection of anti-mycobacterial T-cell memory. Thus, very little is known about the innate immune and IFN-γ-stimulated responses. To explore this, we generated a specific transcriptional signature of the response of human macrophages stimulated in vitro with either Mtb or IFN-γ. These two signatures showed little overlap (Fig. 3 and Supporting Information Fig. 1). Alignment of all significant gene expression differences induced by either stimulus shows that IFN-γ and Mtb induce distinct transcriptional responses, albeit with modest overlap after 24-h Mtb stimulation. The same finding is reflected in the PCA, which shows that changes to transcriptional profiles following innate immune responses to Mtb or stimulation with IFN-γ are evident in different components. Therefore, we used these distinct gene lists to probe the gene expression data from skin samples for innate and IFN-γ-stimulated responses. TST data were subjected to PCA and quantitative differences in each gene signature were represented by the PC1 scores, which reflect the greatest gene expression differences between samples. In this analysis, each set of genes, showed comparable gene expression profiles at 6-h. All 48-h samples clustered separately from 6-h samples, and 48-h samples from TST^+^ subjects showed greater differences than those from TST^−^ subjects (Fig. 4A).

### Immune response amplification in the TST

**Figure 5:**
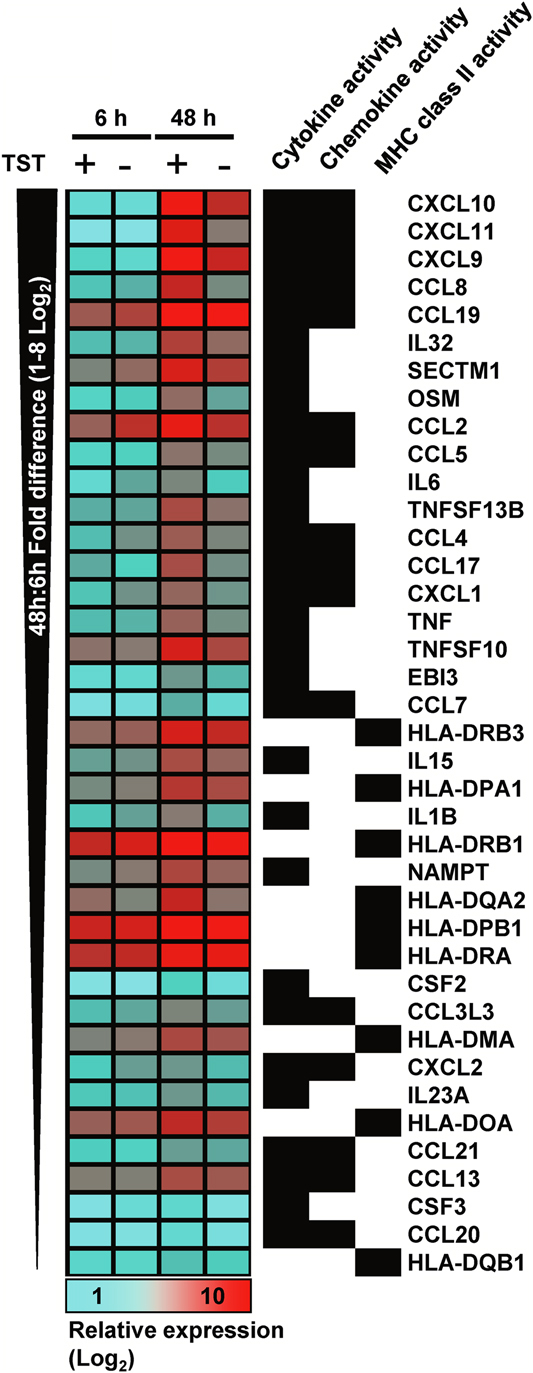
Enriched molecular function gene ontology terms in innate and IFN-γ-stimulated genes within the TST. Mean (relative) expression levels within the TST samples (*N*=4 in each group) are presented for innate immune and IFN-γ-stimulated genes, which showed upregulated expression in 48-h TST and were associated with the top three enriched molecular function gene ontology terms shown.

The innate and IFN-γ-stimulated gene expression signatures associated with development of the TST were analysed further by assessment of gene ontology associations of the genes within each signature, in which expression levels increased (>two-fold) in 48-h samples compared to 6-h samples. Functional annotation clustering analysis revealed that the top three enriched clusters included defense response genes in both data sets, cytokines and chemokines or genes associated with regulation of apoptosis in innate immune signatures (Fig. 4B), and genes associated with antigen presentation or lymphocyte activation in IFN-γ-stimulated signatures (Fig. 4C). Specific analysis of enrichment for gene ontology by molecular function terms of the combined innate and IFN-γ-stimulated genes that were increased within 48-h TST samples, showed striking enrichment for cytokines, and a wide repertoire of chemokines and MHC class II pathway components (Fig. 5). This analysis provides a putative molecular mechanism for robust paracrine amplification of the inflammatory response, mediated by chemokine and cytokine-induced recruitment and activation of lymphocytes, associated with increased MHC class II expression which enhances capacity for antigen presentation. Here again, qualitatively similar but quantitatively smaller changes were evident in TST^−^ subjects.

### Transcriptional regulation of immune responses in the TST

The transcriptional regulation underlying these gene expression changes was investigated by in silico analysis of transcription factor binding motifs in the promoter regions of the genes from innate or IFN-γ signatures that showed increased expression in 48-h TST samples (Fig. 4D). Binding sites for the NF-κB family were the most highly enriched within the innate immune gene signature, whereas binding sites for STAT1 were most highly enriched among the IFN-γ-stimulated genes. These data provide in vivo confirmation of the central roles of NF-κB and STAT1 in transcriptional regulation of innate immune and IFN-γ-stimulated responses respectively.

### Validation of microarray data by qPCR

**Figure 6:**
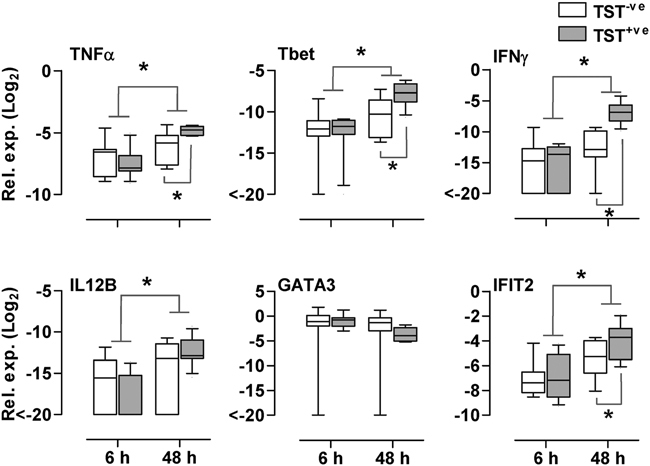
Validation of microarray data by qPCR. Quantitative PCR of selected genes representative of innate immune responses (TNF-α and IL12B), Th1 responses (IFN-γ and Tbet), Th2 responses (GATA3) and IFN-γ-stimulated responses (IFIT2) are shown. Relative gene expression levels are represented in comparison to GAPDH within each sample. Box and whisker plots represent median values, interquartile ranges and 90th percentiles for TST^−^ subjects (*N*=9) and TST^+^ subjects (*N*=11) at each time point. ^*^p<0.05, Mann–Whitney *U* test.

Gene expression patterns identified by microarray analysis in the sample of the study group were validated in all 20 participants (40 samples) by qPCR of selected genes representative of each component of the TST response (Fig. 6). In comparison to gene expression levels in 6-h TST samples, significantly higher levels of innate immune response genes (TNF-α and IL12B), Th1 associated response genes (IFN-γ and Tbet (T-box transcription factor TBX21)) and IFN-γ-inducible genes (IFIT2 (IFN-induced protein with tetratricopeptide repeats 2)) were all evident in all 48-h samples. In each case, gene expression was greater in 48-h samples from TST^+^ subjects compared to TST^−^ subjects, although this did not reach statistical significance for IL12B. In contrast, expression of the transcriptional regulator of Th2 responses did not increase between 6- and 48-h samples and was not significantly different between TST^+^ and TST^−^ subjects, thereby mirroring the microarray data that also showed no evidence for a Th2 component within the TST. The variance in these gene expression data is clearly evident. We found no systematic differences between the UK and SA subjects. In addition, we found no consistent correlation with the magnitude of TST responses clinically, or with IGRA results and no correlation with BCG status. However, the sample size may be insufficient to make firm conclusions and we are extending these studies to explore these correlations further.

## Discussion

The TST has been the cornerstone for in vivo assessments of immune responses to Mtb and is the prototypic example of DTH. In clinical practice, it has been interpreted almost exclusively as a reflection of anti-mycobacterial T-cell memory, although the response is thought to involve interactions between mononuclear phagocytes and T cells. Thus, there has been almost no regard for the role of innate immune and IFN-γ-stimulated responses, and there has also been no qualitative assessment of T-cell responses within tissue microenvironments in humans. There is increasing awareness, however, that such studies are important. In the present study, the application of genome-wide transcriptional profiling of TSTs not only supports many aspects of the existing model of DTH but also provides new insights. It illustrates the potential to probe complex immune responses in vivo at a molecular level and thus achieve better understanding of immunopathogenesis that may inform development of new diagnostic or therapeutic strategies.

Our data are in keeping with the existing model of immunity to mycobacteria, emphasizing the role of Th1 responses, involvement of effector CTLs, and confirming for the first time in human in vivo studies that a Th1 response is associated with increased expression of Tbet, the master Th1 transcriptional regulator 8. In contrast, gene expression signatures for alternatively polarized or Treg cells are absent. However, Th17 responses in vitro develop over 5–10 days 9, and thus the possible emergence of additional T-cell responses at later time point merits further investigation. The lack of transcriptional evidence for cellular proliferation suggests that these responses are due to T-cell recruitment. Local expansion of antigen-specific cells is not a component of the evolving immune response over this time scale. The proportion of the T-cell response, which is antigen-specific, was not assessed directly in this study, but our findings are consistent with data from skin blister models used to study DTH responses to tuberculin, showing little T-cell proliferation or antigen-specific responses at 48–72 h 10.

We show for the first time that the TST harbours specific gene expression signatures for in vivo innate immune responses, invoked by stimulation of macrophages with live Mtb, and IFN-γ-stimulated responses. Equivalent expression of innate immune signatures in TST^+^ and TST^−^ subjects at 6 h suggested that innate immunity was not the critical determinant of the divergence of immune responses at 48 h. Importantly, innate immune responses incorporate multiple homeostatic mechanisms that are involved in the resolution of inflammation 11. Nonetheless, the 48-h time course assessed here showed increased innate immune inflammatory responses in all subjects. In view of the concomitant functionally significant Th1 response, demonstrated by expression of IFN-γ-stimulated gene signatures, and the established role of IFN-γ to support paracrine innate immune amplification 12, the increase in innate immune responses over time is likely to have been driven by the Th1 response.

In this respect, ontological analysis of the innate and IFN-γ-regulated genes within the TST samples showed clear evidence of the two major amplification loops that are pivotal to DTH responses. First, there was significant enrichment of chemokines and cytokines associated with regulating migration of leukocytes into inflamed tissues, the processes which are presumed to drive the formation of the inflammatory infiltrate that is the hallmark of the TST response. Second, there was significant enrichment for genes coding for components of the class II antigen processing and presentation pathway. Lymphocyte recruitment and activation, which then enhances the activity of APCs, can thereby generate robust paracrine amplification of the inflammatory response. Bioinformatic analysis of transcriptional control of the genes involved in these processes provided novel in vivo confirmation of the canonical roles for NF-κB and STAT1 in human innate immune and IFN-γ-stimulated signaling pathways respectively. Further analysis and validation of individual genes within these distinct components of the TST provides an unprecedented opportunity to investigate the molecular details of human anti-mycobacterial immune responses specifically and DTH responses more generally, as they actually occur in vivo.

Transcriptional profiling has provided new qualitative and quantitative measurements of the TST. We have shown that the TST is a multifaceted immune response involving Th1-polarized cells, CTLs, and both innate immune and IFN-γ-stimulated responses, which can be evaluated reproducibly at a molecular level in the human tissue microenvironment. Conventional evaluation of the TST has not provided a consistent measure of susceptibility to TB, and the data presented here do not address this directly, but we were particularly interested to see clear evidence for immunological responses in 48-h samples from TST^−^ subjects. This finding implies that gene expression profiling provides significantly more sensitive assessment of the TST than previous clinical or histological studies. Our results show the potential to use gene expression profiling to obtain valuable immunological data from subjects who have clinically negative TSTs, and hence extend the research applications of this test. In the present study, there were only quantitative differences in otherwise similar immune responses between TST^+^ and TST^−^ subjects. Although some subjects may be exhibiting a primary immune response, the evolution of the response was qualitatively similar in all subjects. Whether TST skin test anergy and discordance between peripheral blood and skin tests might be due to quantitative or qualitative differences in immune responses is of particular interest. Therefore, we propose that transcriptional profiling of TSTs can be applied to broaden our understanding of changes in human immunity to mycobacteria associated with diverse causes, including malnutrition or ageing, immunosuppressive therapies, co-infection with HIV or helminths and in sarcoidosis, in order to obtain new insights in the immunological correlates of protection and pathogenicity in TB.

## Materials and methods

### TSTs

HIV seronegative healthy volunteers were recruited from University College London (UK) and the University of Cape Town (SA). The study was approved by research ethics committees of both institutions and written informed consent was obtained from all participants. TSTs were performed by intradermal injection of 2U Tuberculin (Serum Statens Institute) into each forearm. Two 3-mm punch skin biopsies were taken at one site after 6 h, and two further biopsies taken after clinical assessment at the contralateral site at 48 h. Biopsies were collected into RNAlater (Qiagen) for RNA extraction, or snap frozen for histological analysis. A positive TST was defined clinically as induration >10 mm in diameter at 48 h. TST^+^ patients were evaluated according to local clinical practices in participating UK and SA centres to ensure that they did not have active tuberculosis.

### Mtb-specific IFN-γ release assays

Peripheral blood samples were obtained for QuantiFERON-TB Gold (Cellestis) in UK samples and T-Spot.TB (Oxford Immunotec) tests in SA samples, according to manufacturers' instructions. In samples with adequate numbers of peripheral blood mononuclear cells, responses to 10 μg/mL Mtb purified protein derivative stimulation were also assessed in the ELISpot assays.

### Histological assessment

Ten-micrometer frozen sections were stained with haemotoxylin and eosin and viewed with a Zeiss Axioplan 2 microscope. Digital images were acquired with Axiophot software (version 1.2) and are presented without any subsequent processing. The presence or absence of an inflammatory infiltrate was recorded blind.

### Monocyte-derived macrophage (MDMs) in vitro stimulation experiments

MDM differentiated with macrophage–colony stimulating factor were generated as described previously 13 and stimulated for 4–24 h, with Mtb (H37Rv) at a multiplicity of infection of one, or with 10 ng/mL IFN-γ (Peprotech).

### Transcriptional profiling by cDNA microarray

Total RNA from skin biopsies was obtained with the Lipid Tissue RNEasy kit (Qiagen), and from MDM lysates collected in TRIzol (Invitrogen), with the PureLink RNA kit (Invitrogen). Samples were processed for Agilent microarrays and data were normalized as previously described 14, 15. PCA was used to compare global gene expression profiles and t tests were used to identify significant gene expression differences between samples. DAVID functional annotation clustering (http://david.abcc.ncifcrf.gov) was used to annotate gene lists of interest by gene ontology associations. Transcriptional regulation of specific gene signatures was assessed by analysis of single transcription factor binding site enrichment analysis (http://www.cisreg.ca/cgi-bin/oPOSSUM/opossum). Microarray data are available in the ArrayExpress database (http://www.ebi.ac.uk/arrayexpress) under accession number (E-TABM-1157).

### Quantitative PCR detection of gene transcription

First strand cDNA was synthesized using the qScript cDNASupermix kit (Quanta BioSciences) and quantitative (q)PCR of selected genes was performed as previously described 15 using the following TaqMan inventoried assays: TNFα (Hs00174128_m1), IFN-γ (Hs00174143_m1), Tbet (Hs00894392_m1), IL12B (Hs99999037_m1), GATA3 (GATA binding protein 3) (Hs00231122_m1) and IFIT2 (Interferon-induced protein with tetratricopeptide repeats 2) (Hs00533665_m1).

## Abbreviations

DTH: delayed-type hypersensitivity
MDM: monocytederived macrophage
Mtb: *Mycobacteriumtuberculosis*
PC: principal component
PCA: principal component analysis
TB: tuberculosis
Tbet: T-box transcription factor TBX21
TST: tuberculin skin test
