# Supplementary material for: Transcriptional profiling of innate and adaptive human immune responses to mycobacteria in the tuberculin skin test
Source: Eur J Immunol. 2011 Jul 29;41(11):3253–60. doi: 10.1002/eji.201141841 (PMC3258543; doi:10.1002/eji.201141841)
Supplement: Supplementary file 1 [file eji0041-3253-SD1.pdf]

European Journal of Immunology

Supporting Information

for

DOI 10.1002/eji.201141841

Transcriptional profiling of innate and adaptive human immune responses to myco-bacteria in the tuberculin skin test

Gillian S. Tomlinson, Tamaryn J. Cashmore, Paul T. G. Elkington, John Yates, Rannakoe J. Lehloenya, Jhen Tsang, Michael Brown, Robert F. Miller, Keertan Dheda, David R. Katz, Benjamin M. Chain and Mahdad Noursadeghi

Supplementary Figure 1

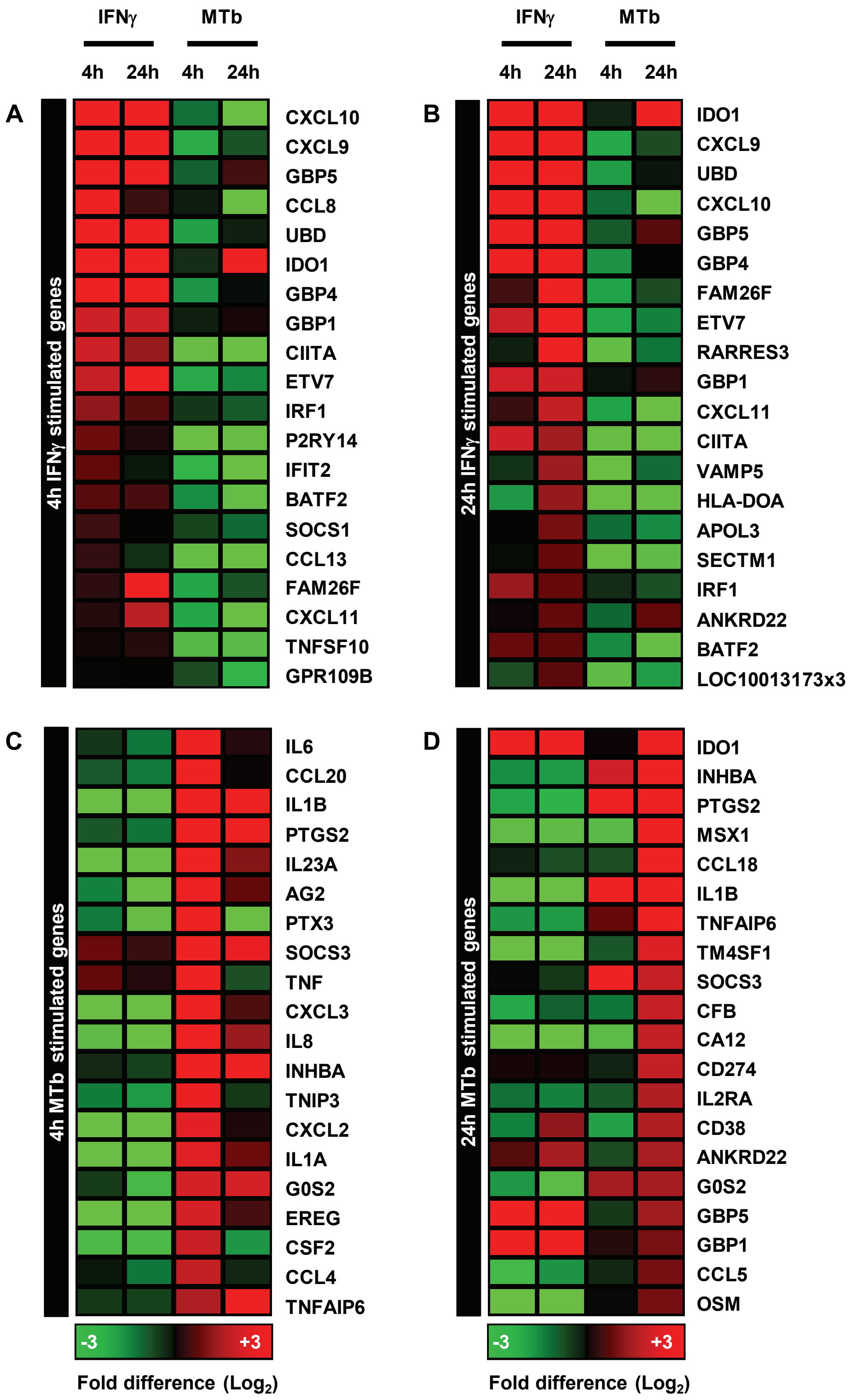

Mtb & IFNγ-stimulated genes in macrophages

Gene expression changes (mean fold difference to unstimulated macrophages from eight different donors) for data sets derived from Mtb and IFNγ stimulated macrophages from three donors are compared for each of the top 20 gene expression changes induced by 4-24h Mtb or IFNγ stimulation. These data show that innate immune responses to Mtb and IFNγ stimulated changes show limited overlap.

Supplementary Table 1: Study Subjects Demographic Data

Study ID	Gender	Age	Ethnicity	BCG	Previous TB	TST (mm)	Histological Inflammation	QuantiFERON	T-SPOT.TB	PPD ELIspot
V1*	F	26	White	✗	✗	11	✓	Negative	Negative	Negative
V2*	M	22	White	✓	✗	11	✓	Negative	Negative	N/A
V3	M	22	White	✗	✗	0	✗	Negative	Negative	N/A
V4	F	33	White	✓	✗	0	✗	Negative	Negative	Negative
V5	F	38	Black	✗	✗	10	✓	Negative	Negative	N/A
V6	F	40	White	✗	✗	0	✗	Negative	Negative	Positive
V7*	M	22	Mixed	✗	✗	0	✗	Negative	Negative	Positive
V8*	M	32	White	✗	✗	0	✗	Negative	Negative	Positive
CT1*	F	49	White	N/A	✗	24	✓	N/A	Negative	N/A
CT2*	M	37	Black	N/A	✗	21	✓	N/A	Positive	N/A
CT3	F	35	Black	N/A	✓	35	✓	N/A	Negative	N/A
CT4*	F	44	Mixed	N/A	✗	0	✗	N/A	Negative	N/A
CT5	M	43	White	N/A	✗	0	✓	N/A	Negative	N/A
CT6	M	41	Black	N/A	✗	35	✓	N/A	Negative	N/A
CT7	F	51	Mixed	N/A	✗	25	✓	N/A	Negative	N/A
CT8	F	39	Mixed	N/A	✗	24	✓	N/A	Positive	N/A
CT9*	F	36	Mixed	N/A	✗	0	✗	N/A	Negative	N/A
CT10	F	31	White	N/A	✗	0	✗	N/A	Negative	N/A
CT11	F	39	Mixed	N/A	✗	0	✗	N/A	Negative	N/A
CT12	F	44	Mixed	N/A	✗	52	✓	N/A	Positive	N/A

***Samples analysed by microarrays obtained from these subjects**
